# Compressed professionalization in informal economies: a socio-technical analysis of youth-led artificial intelligence adoption in the Democratic Republic of the Congo

**DOI:** 10.3389/frai.2026.1868452

**Published:** 2026-06-03

**Authors:** Delphin B. Kyubwa

**Affiliations:** School of Engineering, Université Technologique du Congo (UTC-Uvira), Uvira, Democratic Republic of Congo

**Keywords:** artificial intelligence (AI), compressed professionalization, informal economies, ICT4D, socio-technical systems, youth innovation, Democratic Republic of the Congo (DRC), AI governance

## Abstract

Artificial intelligence (AI) is increasingly shaping development trajectories across the Global South, yet limited attention has been paid to how AI is appropriated within highly informal and institutionally fragile economies. This article advances a conceptually driven, theory-building analysis supported by qualitative field insights used as illustrative grounding rather than for statistical generalization. Drawing on 125 semi-structured interviews conducted in Kinshasa, Lubumbashi, and Goma, and integrating Information and Communication Technologies for Development (ICT4D), socio-technical systems theory, and the capability approach, the study examines how infrastructural constraints, fragmented governance, and uneven skill ecosystems interact with youth-driven innovation to shape AI adoption in the Democratic Republic of the Congo (DRC). The article introduces compressed professionalization, defined as the accelerated acquisition and immediate market enactment of professional-level digital capabilities outside formal institutional pathways. Empirical observations show that youth mobilize AI tools for translation, content creation, customer engagement, and micro-entrepreneurial activities, enabling partial and situational approximation of selected formal-sector practices. The analysis further conceptualizes AI as a conditional capability amplifier, expanding agency while producing uneven inclusion shaped by disparities in connectivity, skills, and infrastructure. Rather than following policy-led or infrastructure-first trajectories, AI adoption emerges through hybrid socio-technical interactions between bottom-up youth innovation and weakly coordinated institutional frameworks. The article concludes by proposing a Strategic Action Framework to support more inclusive and context-responsive AI ecosystems. While grounded in the DRC, the findings offer broader insights into AI adoption dynamics across informal economies in Sub-Saharan Africa and beyond.

## 1 Introduction

### 1.1 Background and rationale

Artificial intelligence (AI) has become central to development debates across the Global South, yet empirical evidence on how AI is adopted within highly informal and institutionally fragile economies remains limited (Gwagwa et al., 2022; Arakpogun et al., 2021). In the Democratic Republic of the Congo (DRC), recent policy initiatives signal growing political interest in AI, but implementation unfolds within a context marked by infrastructural scarcity, fragmented governance, and a predominantly informal economy (Bánhidi and Dobos, 2024; Foster and Heeks, 2013).

Existing research on AI in Africa has largely focused on national strategies, governance frameworks, or sector-specific pilot projects, offering limited insight into how AI technologies are appropriated in everyday economic practices or how youth navigate structural constraints within informal markets (Heeks, 2022; Walsham, 2017). As a result, the lived dynamics of AI adoption in contexts characterized by pervasive informality and weak institutional mediation remain underexplored. Earlier work examining digital technologies in the Congolese context has highlighted the importance of situating digital adoption within local socio-economic and institutional realities (Kyubwa, 2020).

Within this constrained environment, youth have emerged as central actors in digital experimentation. Young entrepreneurs increasingly deploy AI-enabled tools for translation, content creation, customer engagement, and micro-automation, primarily through mobile platforms and social media ecosystems. This mobile-first pattern of AI use mirrors broader regional connectivity dynamics (GSMA, 2023). Such practices align with wider patterns of frugal and grassroots innovation across Africa, in which digital technologies are creatively repurposed to compensate for institutional and infrastructural limitations (Radjou, 2020).

Beyond their immediate functional benefits, these AI practices shape youth economic resilience and longer-term development trajectories in fragile contexts, particularly in the absence of stable formal employment and effective institutional support. AI adoption in informal economies should therefore be understood not merely as technological uptake, but as a socio-technical process through which youth negotiate pathways toward sustainable livelihoods, inclusive development, and incremental capacity-building under conditions of structural constraint.

At the policy level, national frameworks such as the *Plan National du Numérique 2026–2030* articulate ambitious objectives for digital transformation. However, implementation remains constrained by high connectivity costs, unreliable electricity, shortages of specialized digital skills, and fragmented governance arrangements (International Telecommunication Union, 2024; Foster and Heeks, 2013). This disconnect between policy ambition and everyday practice underscores the need for empirical analysis that foregrounds bottom-up adoption pathways alongside formal institutional frameworks.

This article addresses this gap by developing a conceptual and socio-technical account of youth-led AI adoption in the informal economy of the DRC through multi-site qualitative fieldwork. It asks: How do youth in the DRC appropriate AI technologies under conditions of infrastructural scarcity, governance fragmentation, and pervasive informality?

To answer this question, the study integrates ICT4D scholarship, socio-technical systems theory, and the capability approach to analyze how technology, institutions, and human agency interact under conditions of structural constraint.

Conceptually, this study advances a socio-technical account of AI adoption in which youth-driven innovation contributes to a process we term *Compressed Professionalization*. Generative AI tools enable youth in informal economies to rapidly acquire and apply professional digital skills, allowing them to partially and situationally approximate selected formal-sector practices without conventional institutional pathways. We define *Compressed Professionalization* as the accelerated acquisition and deployment of professional-level digital capabilities outside traditional training structures, mediated by AI-enabled access to knowledge, tools, and digital platforms.

### 1.2 Theoretical positioning

This study integrates three complementary theoretical perspectives—ICT4D, socio-technical systems theory, and the capability approach—to examine AI adoption in fragile and highly informal contexts. Together, these lenses enable an analysis of how technology, institutions, and human agency interact under conditions of structural scarcity.

ICT4D scholarship emphasizes that technological outcomes in the Global South are shaped less by availability than by contextual, institutional, and infrastructural conditions (Heeks, 2022; Walsham, 2017). Technologies do not produce development outcomes automatically; rather, they are mediated by inequalities, governance arrangements, and socio-economic realities (Kleine, 2010).

Socio-technical systems theory highlights the co-construction of technology through interactions among users, technological artefacts, and institutional environments, foregrounding adaptation and embeddedness in specific social contexts (Avgerou, 2008; Orlikowski and Iacono, 2001; Bijker, 1995).

The capability approach conceptualizes technology as a means of expanding agency, skills, and livelihood opportunities (Sen, 1999; Alkire, 2002). Applied here, it suggests that informal AI practices function as capability-expanding mechanisms for urban youth, enabling new forms of economic participation despite persistent constraints.

Taken together, these perspectives position AI adoption as a situated and co-constructed process, shaped by governance, infrastructure, skills, and localized innovation, rather than a linear or technology-driven pathway.

### 1.3 What this study contributes

This study introduces Compressed Professionalization, defined as the accelerated acquisition and immediate market deployment of professional-level digital capabilities, enabled by AI, outside formal institutional pathways. Unlike existing notions of digital upskilling or informal learning, this concept emphasizes the speed and performative enactment of capability, whereby individuals not only acquire skills but rapidly operationalize them in market-facing contexts without formal institutional validation (Heeks, 2022; Kleine, 2010).

In this sense, compressed professionalization foregrounds a shift from learning to immediate application, highlighting how individuals convert emerging competencies into economically relevant outputs in real time, often mediated through digital platforms and informal learning ecosystems (Radjou, 2020; Walsham, 2017).

This distinguishes compressed professionalization from broader notions of digital inclusion or gig work by prioritizing accelerated capability enactment over mere labor participation (Sen, 1999). In this sense, the concept captures a transition from access and participation toward the rapid production and demonstration of professional-level outputs within informal and resource-constrained environments (Avgerou, 2008; Toyama, 2015).

Building on this conceptual foundation, the article advances four interrelated theoretical contributions to ICT4D and socio-technical systems scholarship. First, it introduces compressed professionalization as a mechanism explaining how AI enables youth in informal economies to partially and situationally approximate selected formal-sector practices without conventional institutional transitions, resulting in partial and uneven capability formation (Sen, 1999; Heeks, 2022; Meagher, 2018; Avgerou, 2008).

Second, the article advances a two-pathway model of AI adoption that distinguishes bottom-up, youth-led innovation from top-down policy structures, highlighting persistent misalignments between grassroots experimentation and institutional frameworks (Walsham, 2017; Heeks, 2022).

Third, it conceptualizes AI as a conditional capability amplifier, expanding youth agency while simultaneously producing stratified inclusion shaped by differential access to connectivity, skills, and infrastructure (Sen, 1999).

Finally, the article extends ICT4D theory beyond infrastructure-first models by demonstrating that AI adoption in highly informal economies is shaped by socio-technical configurations rather than access alone (Avgerou, 2008; Orlikowski and Iacono, 2001).

### 1.4 Conceptual positioning and contribution to AI and society

This article is positioned as a conceptually driven, integrative analysis that draws on empirical insights to advance a broader theoretical argument about AI adoption in informal economies. It adopts a theory-building approach in which qualitative field data are used as illustrative grounding for conceptual development.

In line with the aims of *AI & Society*, the analysis moves beyond descriptive accounts of technological uptake to critically examine how AI reshapes relationships between knowledge, work, and institutional structures under conditions of structural constraint (Avgerou, 2008; Walsham, 2017). Rather than reiterating dominant themes such as regulation or transparency, the article mobilizes *compressed professionalization* (see Section 1.3) as its central analytical lens. It explains emerging forms of capability formation and socio-economic participation (Heeks, 2022; Sen, 1999).

Within this framework, empirical materials serve as illustrative evidence for conceptual refinement rather than as a basis for statistical generalization, enabling a bridge between empirical observation and theoretical synthesis consistent with socio-technical and ICT4D traditions. The article contributes to debates on inclusive and context-sensitive AI governance by foregrounding youth agency and informal innovation practices (Gwagwa et al., 2022; Plantinga et al., 2024). Integrating socio-technical and capability-based perspectives, it presents AI as a socially embedded and unevenly distributed phenomenon (Orlikowski and Iacono, 2001; Kleine, 2010), aligned with the journal’s emphasis on critical and theoretically grounded analysis.

*This article makes three core contributions*. First, it introduces the concept of *compressed professionalization* to explain how generative AI enables the accelerated formation and immediate enactment of professional-level capabilities outside formal institutional pathways. Unlike existing notions of digital upskilling or platform labor, this concept captures a shift from gradual learning to the real-time deployment of capabilities under conditions of constraint.

Second, the article develops a process-based account of AI as a *conditional capability amplifier*, specifying mechanisms—such as cognitive scaffolding, iterative feedback, and reduced learning-to-application lag—through which AI reshapes skill formation and economic participation.

Third, it advances a non-linear, socio-technical model of AI adoption in informal economies, showing that bottom-up innovation can precede and outpace institutional alignment. In doing so, the study bridges ICT4D and AI scholarship, offering a theoretically grounded account of how generative AI is transforming the relationship between knowledge, work, and capability in resource-constrained environments.

## 2 Conceptual framework and literature review

The DRC is characterized by persistent infrastructural constraints, institutional fragmentation, and a large, youth-dominated informal economy. These conditions shape how digital technologies, including AI, are accessed, adapted, and appropriated, positioning the DRC as a critical case for examining AI adoption beyond formal innovation ecosystems. Debates on digital industrialization further situate AI adoption within broader trajectories of structural transformation in low and middle-income economies (United Nations Conference on Trade and Development, 2022).

These dynamics must also be situated within broader critical debates on platform dependency, data colonialism, and uneven global power structures shaping AI systems. As most AI technologies are developed outside the Global South, their adoption may reproduce asymmetries in knowledge production, value extraction, and technological control, even as they expand local capabilities.

### 2.1 Conceptual foundation

This article is positioned as a *conceptually driven empirical study* that uses qualitative fieldwork as illustrative grounding for theoretical development. Scholarship on digital transformation emphasizes that the diffusion and societal impact of emerging technologies depend on the alignment of infrastructure, human capital, and institutional frameworks (Brynjolfsson and McAfee, 2014; Schwab, 2016). From an inclusive innovation perspective, development outcomes are shaped by the extent to which marginalized groups—particularly youth—can access, adapt, and appropriate technological opportunities under conditions of structural constraint (Sen, 1999; Radjou, 2020). Complementing this view, research on AI governance underscores the importance of ethical, adaptive, and context-sensitive regulatory frameworks, especially in low-resource and institutionally fragile environments (United Nations Educational, Scientific, and Cultural Organization, 2021; Plantinga et al., 2024).

While existing studies document Africa’s infrastructural and institutional challenges (Kazim, 2021; United Nations Economic Commission for Africa, 2020), they provide limited insight into how these conditions translate into concrete patterns of AI adoption within informal economies. To address this gap, this study conceptualizes AI as a *socio-technical lever* shaped by the interaction of skills development, governance arrangements, and frugal, youth-driven innovation practices. In doing so, it advances a more grounded understanding of how AI adoption emerges in contexts characterized by high informality, weak institutional mediation, and acute resource constraints.

### 2.2 A socio-technical, youth-centered framework

Drawing on empirical data from 125 interviews and established theory, the study develops a four-pillar socio-technical framework identifying the conditions shaping AI adoption:

1. *Infrastructure*: digital access, affordability, and energy reliability
2. *Governance*: policy coherence, regulatory capacity, and institutional coordination
3. *Skills*: formal, non-formal, and peer-based learning pathways
4. *Innovation*: youth creativity, frugal solutions, and micro-entrepreneurship.

These pillars serve as the core analytical lenses guiding the interpretation of findings across the Results and Discussion sections. Drawing on ICT4D scholarship, the analysis explains how institutional weaknesses mediate technological outcomes (Heeks, 2022). Socio-technical systems theory is used to clarify the interactions between governance constraints and grassroots digital practices (Avgerou, 2008). The capability approach further illuminates how youth expand agency through self-directed learning and digital entrepreneurship (Sen, 1999; Alkire, 2002).

The initiatives and policy frameworks depicted in the figure are used illustratively to anchor the study’s conceptual argument and do not constitute an exhaustive or comparative assessment of individual programs. Rather, the figure presents a conceptual typology of AI adoption in the DRC’s informal economy, distinguishing two interacting pathways: bottom-up, youth-led innovation and top-down policy and institutional frameworks.

**Figure 1:**
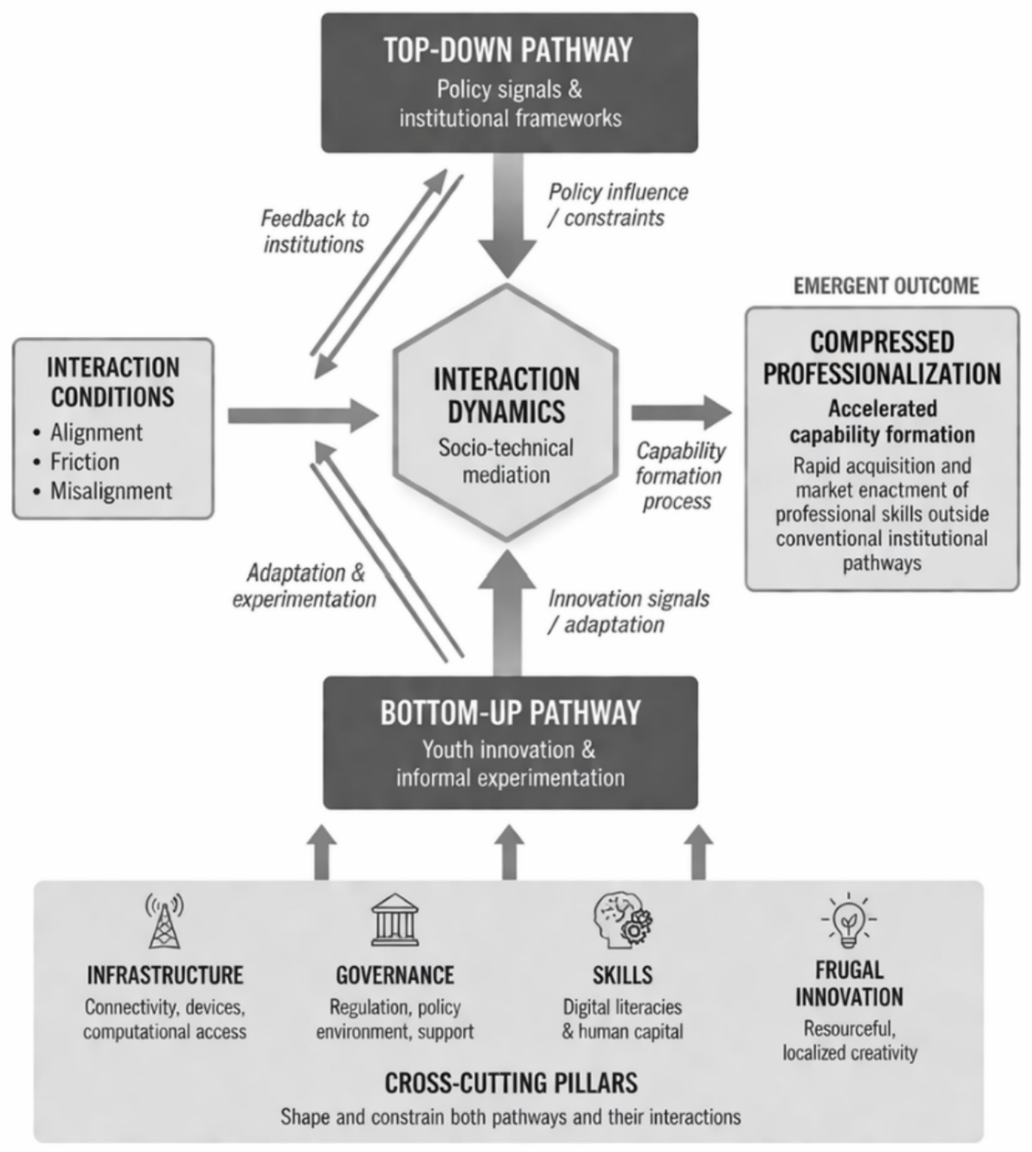
Compressed professionalization: a socio-technical model of AI adoption.

Figure 1 Socio-technical model of AI adoption in informal economies. The model illustrates the dynamic interaction between top-down institutional frameworks and bottom-up youth-led innovation, mediated by socio-technical conditions of alignment, friction, and misalignment. These interactions, shaped by cross-cutting infrastructural, governance, skills, and frugal innovation factors, give rise to compressed professionalization as an emergent outcome of accelerated capability formation.

Compressed professionalization is conceptualized as the process through which young actors rapidly acquire and perform professional capabilities outside formal institutional trajectories. The framework depicts AI adoption as a hybrid socio-technical process shaped by uneven interactions between institutional intent and grassroots practice.

### 2.3 Literature review and theoretical contribution

Building on these perspectives, this article conceptualizes compressed professionalization (as defined above) as a socio-technical process through which AI tools enable individuals in informal economies to acquire and deploy professional capabilities outside traditional institutional pathways rapidly. More specifically, it captures the accelerated acquisition and immediate market enactment of professional-level skills, effectively bypassing conventional upskilling trajectories and formal validation mechanisms (Heeks, 2022; Kleine, 2010).

This framing differentiates compressed professionalization from existing accounts of digital inclusion and platform-based labor by emphasizing capability enactment rather than participation alone (Sen, 1999; Srnicek, 2017). Enabled by AI tools, open online knowledge, and digital platforms, individuals can rapidly develop competencies, produce professional outputs, and engage in economic activities that have historically required prolonged formal education or institutional affiliation (Radjou, 2020; Walsham, 2017).

This conceptualization situates AI adoption within broader debates on digital transformation and development. Research on digital transformation in Africa highlights that effective integration depends not only on infrastructure but also on human capital and institutional capacity (World Bank, 2023; Organisation for Economic Co-operation and Development, 2023; United Nations Conference on Trade and Development, 2022). Recent initiatives, such as the African Union’s Continental AI Strategy, further underscore the need for ethical, inclusive, and interoperable governance frameworks (African Union Commission, 2024).

At the same time, scholarship on frugal innovation demonstrates how resource-constrained environments generate adaptive, localized solutions (Bhatti et al., 2018; Radjou, 2020), though limited attention has been paid to their intersection with AI or to governance conditions shaping their scalability. Across ICT4D and informal economy literatures, institutional fragility remains a central factor shaping digital adoption (Avgerou, 2008; Heeks, 2022; Meagher, 2018). Rather than diffusing primarily through formal policy channels, innovation often unfolds through socially embedded, experimental practices, underscoring the need for an integrated framework capturing the interaction between technology, skills, governance, and informal economic dynamics.

Unlike digital upskilling, which emphasizes gradual skill acquisition and human capital development over time (World Bank, 2023; Organisation for Economic Co-operation and Development, 2023), compressed professionalization foregrounds immediate capability enactment. Unlike gig economy frameworks, which focus primarily on labor participation within platform-mediated markets (Srnicek, 2017; Wood et al., 2019), it emphasizes the rapid formation and deployment of professional-level capabilities. It also differs from informal learning by highlighting not only knowledge acquisition but the accelerated production of market-facing outputs (Eraut, 2004; Marsick and Watkins, 2015). In this sense, the concept captures a shift from participation to performative capability.

### 2.4 Theoretical contribution

This study makes four contributions:

1. It introduces compressed professionalization as a mechanism explaining accelerated capability formation in informal economies.
2. It proposes a two-pathway model distinguishing bottom-up innovation from top-down institutional frameworks.
3. It reframes AI as a conditional capability amplifier, producing uneven inclusion.
4. It extends ICT4D theory beyond infrastructure-first models, emphasizing socio-technical configurations.

More broadly, the study advances ICT4D and AI governance scholarship by conceptualizing AI adoption in highly informal and institutionally fragile economies as a youth-driven socio-technical process, rather than a predominantly policy-led or infrastructure-dependent transition (Avgerou, 2008; Orlikowski and Iacono, 2001; Walsham, 2017). While ICT4D research has emphasized institutions, infrastructure, and human capital, it has often treated AI as an extension of earlier digital technologies (Heeks, 2022). In contrast, the findings show that AI introduces a distinct socio-technical dynamic, functioning as a conditional capability amplifier under conditions of scarcity and enabling youth to bypass traditional barriers related to formal education, organizational membership, and capital intensity (Sen, 1999; Alkire, 2002).

Empirically, the study demonstrates how generative AI enables compressed professionalization within the informal economy, allowing youth to rapidly acquire functional digital skills and partially and situationally approximate selected formal-sector practices without formal institutional transitions (Radjou, 2020; Bhatti et al., 2018; Meagher, 2018). These findings suggest that AI is not merely a productivity tool but an accelerator of professional capability, enabling individuals to perform tasks traditionally associated with formally trained professionals. This dynamic challenges linear models of digital transformation that assume a sequential progression from infrastructure and policy readiness to adoption and impact (Foster and Heeks, 2013). Instead, the study advances a two-pathway model in which bottom-up, youth-led innovation and top-down policy frameworks interact in uneven and often misaligned ways (Avgerou, 2008; Heeks, 2022).

By integrating socio-technical systems theory with the capability approach, the study develops a youth-centered analytical lens that foregrounds agency, improvisation, and informal learning as central mechanisms of AI adoption in weak governance contexts (Orlikowski and Iacono, 2001; Sen, 1999). Overall, it refines ICT4D theory by demonstrating how AI enables compressed professionalization through youth-driven socio-technical adaptation, rather than through infrastructure or policy-first pathways.

## 3 Empirical basis and analytical approach

### 3.1 Research design

The empirical material presented in this article serves primarily as illustrative grounding for conceptual development, consistent with its positioning as a socio-technical review. Accordingly, the study adopts a qualitative, multi-site case study design suited to examining complex socio-technical processes as they unfold in real-world contexts (Yin, 2018). Case study approaches are widely used in ICT4D research to investigate how digital technologies interact with institutional, infrastructural, and cultural conditions (Walsham, 2017; Heeks, 2022).

In the Democratic Republic of the Congo—characterized by a highly informal economy and fragmented governance structures—this qualitative design is particularly appropriate for capturing the lived experiences, adaptive strategies, and innovation practices of youth operating under structural constraints.

The analysis draws on three complementary sources of empirical material: (1) 125 semi-structured interviews with youth entrepreneurs, digital workers, policymakers, and ecosystem actors; (2) documentary analysis of national strategies, regulatory frameworks, and sectoral reports; and (3) a drivers-and-barriers synthesis used to consolidate enabling and constraining factors shaping AI adoption. This triangulated approach enhances the credibility, robustness, and interpretive depth of the analysis (Creswell and Plano Clark, 2018) and aligns with participatory and user-centered traditions in ICT4D research (Byrne and Sahay, 2007).

### 3.2 Study sites

Data collection took place in Kinshasa, Lubumbashi, and Goma—three urban centers representing distinct socio-economic, infrastructural, and institutional conditions.

- *Kinshasa* is the political and economic capital, with the highest concentration of digital services, universities, and innovation hubs.
- *Lubumbashi* is a mining-driven city characterized by growing private-sector investment and a relatively more structured entrepreneurial ecosystem.
- *Goma* functions as a humanitarian and cross-border trade hub, marked by a strong NGO presence and high exposure to donor-supported digital skills programs.

These sites were selected to capture variation in infrastructure availability, governance presence, and youth innovation ecosystems, enabling comparative insights across urban contexts.

Participants were recruited in Kinshasa, Lubumbashi, and Goma through a combination of snowball sampling, referrals from youth innovation networks, and outreach via local training centers and informal digital communities. While this approach facilitated access to active AI users, it may over represent digitally engaged youth relative to the broader informal economy. To mitigate this risk, recruitment deliberately included participants with varying levels of technical proficiency and AI use intensity, including those experimenting with AI tools intermittently or at a basic level.

### 3.3 Sampling strategy

The study employed purposive sampling to identify youth (ages 18–35) engaged in digital or AI-related activities, including content creators, translators, digital marketers, small-scale e-commerce operators, agricultural micro-entrepreneurs using AI-enabled tools, and students or recent graduates experimenting with AI. This approach is appropriate for exploratory ICT4D research aimed at understanding emerging practices rather than achieving statistical representativeness (Etikan et al., 2016).

To ensure diversity across urban contexts, the sample included 55 participants from Kinshasa, 40 from Lubumbashi, and 30 from Goma. Participants were recruited through innovation hubs, WhatsApp groups, university networks, and snowball referrals. This strategy effectively captures early-stage AI adoption, which remains concentrated among digitally engaged youth within informal innovation networks. The sample size (*n* = 125) is consistent with qualitative saturation in multi-site ICT4D research.

### 3.4 Data collection

#### 3.4.1 Semi-structured interviews

Interviews lasted between 45 and 90 min and explored participants’ digital practices and AI use; skill acquisition pathways; infrastructural and financial constraints; perceptions of governance and regulation; and innovation strategies and business models. Semi-structured interviews enabled participants to articulate their experiences in their own terms while allowing for thematic comparability across sites and participant groups (Kvale and Brinkmann, 2015). Participants reported using a range of AI tools, including large language models (e.g., ChatGPT), image generation systems, and chatbot-based applications, providing insight into the practical contexts of AI adoption.

#### 3.4.2 Documentary analysis

The documentary analysis encompassed national, regional, and international policy and regulatory sources relevant to digital governance and AI adoption. Reviewed documents included the *Plan National du Numérique—Horizon 2025* (Ministère du Numérique, 2020), alongside policy materials outlining the subsequent 2026–2030 digital strategy phase, as well as regulatory and market observatory reports issued by the Autorité de Régulation de la Poste et des Télécommunications du Congo (ARPTC, 2023). Additional institutional context was drawn from the African Union Continental AI Strategy (2024) and digital economy assessments published by the International Telecommunication Union, the Organisation for Economic Co-operation and Development, and the World Bank. Together, these sources provided institutional context and supported the triangulation of interview findings.

### 3.5 Data analysis

Data were analyzed using thematic coding in NVivo, following a hybrid inductive–deductive approach (Miles et al., 2014). Deductive codes were derived from the four socio-technical pillars guiding the analysis: infrastructure, governance, skills, and innovation, while inductive codes emerged from participants’ narratives. These inductive codes captured recurrent themes such as AI as a shortcut, data costs as a barrier, peer learning, WhatsApp as a training space, electricity constraints, and the use of AI to build customer trust.

The coding process proceeded in three stages. First, open coding was used to identify initial concepts across the dataset. Second, axial coding was applied to establish relationships between categories and sub-themes. Third, selective coding integrated the findings into the overarching socio-technical framework, enabling systematic interpretation across cases.

### 3.6 Coding reliability and analytic rigor

Analytic rigor and credibility were ensured through iterative codebook development, repeated coding, and the systematic use of analytic memos to document coding decisions, emerging patterns, and theoretical reflections (Miles et al., 2014). An initial codebook was developed from a subset of interviews and refined across multiple coding cycles, with stability reinforced through re-coding at different stages of analysis.

Given the study’s interpretive and conceptually driven orientation, emphasis is placed on analytic depth, reflexivity, and theoretical coherence rather than on statistical measures of inter-coder reliability. This approach aligns with qualitative ICT4D traditions, which prioritize contextual interpretation, triangulation, and iterative validation of themes over formal reliability metrics.

Although primary coding was conducted by the author, peer debriefing with academic colleagues was used to review code definitions, challenge interpretations, and refine thematic boundaries (Walsham, 2017). Credibility was further strengthened through triangulation across interview data, study sites, participant profiles, and policy documents, consistent with qualitative case study practices (Creswell and Plano Clark, 2018; Yin, 2018). Themes were validated through constant comparison and assessment of their recurrence and explanatory coherence across the dataset.

### 3.7 Researcher positionality

The researcher occupies a hybrid insider–outsider position, enabling access to participants while necessitating critical reflexivity to mitigate potential interpretive bias. This positionality facilitated trust and engagement in the field, while also requiring careful attention to avoid over-interpretation of narratives through policy-oriented lenses (Von Unger, 2021). Reflexive memos were maintained throughout data collection and analysis to support ongoing critical self-examination and enhance analytic rigor.

### 3.8 Ethical considerations

Ethical approval was obtained from relevant institutional bodies. All participants provided informed consent, and pseudonyms were used to protect confidentiality, in line with established qualitative research ethics (Kvale and Brinkmann, 2015). Given the sensitivity of discussing informal economic activities, interviews were conducted in participant-chosen locations to minimize risk and power asymmetries (Von Unger, 2021). Data were securely stored and anonymized during analysis in accordance with standard ethical guidelines for ICT4D research (Walsham, 2017).

### 3.9 Qualitative data analysis and coding transparency

Data were analyzed using a hybrid inductive–deductive thematic coding approach implemented in NVivo, enabling systematic organization and comparison of themes across interviews, study sites, and participant profiles (Miles et al., 2014).

To enhance methodological transparency, the coding process is illustrated with a concrete example. One participant explained:

*I use a WhatsApp bot when I have data. I prepare messages in advance and send them quickly to customers before the connection drops. It saves time, but I cannot use it every day because data is expensive.*

This excerpt was initially coded inductively as intermittent AI use, mobile-first adaptation, and data cost constraint. During axial coding, these inductive codes were grouped under the category *frugal AI practices*, which was subsequently mapped to the deductive analytical pillar *Bottom-Up Innovation*. This iterative process ensured that analytical categories remained empirically grounded while being theoretically anchored within the socio-technical framework.

### 3.10 Methodological limitations

This study focuses on urban youth engaged in digital and AI-related activities and may therefore underrepresent rural populations and informal actors with limited or no access to digital technologies. Consequently, the findings illuminate early-stage and emergent patterns of AI adoption rather than national diffusion dynamics. The qualitative design prioritizes depth and contextual understanding over statistical generalizability, consistent with the exploratory aims of ICT4D research.

## 4 Empirical insights

As illustrated in Figure 1, AI adoption in the Congolese informal economy emerges from a persistent tension between top-down digital policy frameworks and bottom-up, youth-led innovation pathways. While the *Plan National du Numérique 2026–2030* emphasizes formal training programs, institutional capacity building, and standardized digital skills pathways, youth AI adoption relies predominantly on informal, peer-based learning ecosystems, including WhatsApp groups, YouTube tutorials, and experiential experimentation. This disconnect constrains the relevance and reach of national policy initiatives, which remain weakly aligned with the decentralized and adaptive learning modalities through which youth currently acquire AI-related skills.

The empirical analysis suggests that AI adoption is fundamentally a youth-driven phenomenon, unfolding not as a linear progression from policy to practice but through the friction and partial alignment between the two pathways depicted in Figure 1. The analysis is organized around four socio-technical pillars—infrastructure, governance, skills, and innovation—which serve as the primary conduits through which bottom-up innovation and top-down policy interact. Examining these pillars reveals where limited zones of alignment support AI integration and where structural barriers lead the pathways to diverge.

### 4.1 Infrastructure: access, affordability, and reliability

Infrastructure emerged as the most significant constraint shaping AI adoption.

#### 4.1.1 High data costs

Participants consistently described mobile data costs as prohibitive. As one youth entrepreneur in Kinshasa explained: “AI helps me work faster, but the internet costs more than what I earn in a day.” This aligns with ITU (2024) findings that the DRC has among the highest data-cost-to-income ratios in Africa.

#### 4.1.2 Unreliable electricity

Participants frequently identified electricity shortages as a major constraint on AI use, particularly for activities requiring sustained connectivity and device charging. Interruptions were described as unpredictable and disruptive to both learning and income-generating tasks. As one participant in Goma explained, “When the power goes, everything stops. You can’t rely on AI if you can’t rely on electricity.”

#### 4.1.3 Mobile-first AI adoption

Participants reported that AI use was predominantly mobile-based due to limited access to laptops and desktop computers. Most interactions with AI tools occurred through:

- smartphones
- WhatsApp-based chatbots
- lightweight mobile applications

As one respondent in Lubumbashi explained, “Everything I do with AI is on my phone. I don’t have a computer, but my phone is enough to work and learn.”

### 4.2 Governance: fragmentation, uncertainty, and emerging frameworks

#### 4.2.1 Fragmented institutional coordination

Participants frequently expressed confusion regarding institutional responsibility for AI governance. Many were aware of the existence of national strategies but reported uncertainty about which institutions were responsible for implementation or oversight.

As one digital worker in Lubumbashi stated, “We hear about strategies, but no one knows who is responsible for what.”

#### 4.2.2 Regulatory ambiguity

Regulatory ambiguity persists despite recent oversight efforts by national regulators (ARPTC, 2023). Youth participants consistently reported uncertainty surrounding key regulatory issues affecting their digital activities, including data protection requirements, the use and ownership of AI-generated content, and the registration and formalization of digital businesses.

This uncertainty was widely perceived as a constraint on growth and experimentation, shaping cautious and short-term innovation strategies. As one content creator in Kinshasa noted, “We want to grow, but we don’t know the rules.”

#### 4.2.3 Policy aspirations and implementation gaps

Participants acknowledged the presence of national policy ambitions related to digital transformation and AI but reported limited evidence of concrete implementation in their daily activities. Several respondents noted that policy announcements were not accompanied by visible support mechanisms, coordination, or resources at the local level.

As one participant in Goma observed, “They talk about digital development, but on the ground, nothing really changes for us.”

### 4.3 Skills: informal learning, peer networks, and capability expansion

#### 4.3.1 Informal learning ecosystems

Youth participants reported relying primarily on informal and peer-based learning channels to acquire AI-related skills. The most frequently cited resources included:

- YouTube tutorials
- WhatsApp groups
- peer-to-peer mentoring

As one student in Goma explained, “We learn from each other. No one teaches AI in school.”

Participants also emphasized the role of AI tools in accelerating skill acquisition through experimentation. As one respondent in Kinshasa noted, “With AI tools, I can do in one day what used to take me weeks to learn. I don’t need formal training anymore; I just experiment and improve as I go.”

#### 4.3.2 Limited formal training

Universities offer few AI-related courses, and private training programs are expensive. Participants described a “skills gap” between policy aspirations and educational realities.

#### 4.3.3 Rapid experimentation

Despite limited training, youth experiment with:

- ChatGPT
- Midjourney style tools
- Translation models
- AI-assisted design apps

This experimentation reflects frugal-innovation patterns (Radjou, 2020).

### 4.4 Innovation: youth creativity, micro-entrepreneurship, and emerging business models

#### 4.4.1 AI-enabled micro-entrepreneurship

Youth use AI to:

- Design logos
- Translate documents
- Automate customer responses
- Generate marketing content
- Manage inventory

A young entrepreneur in Lubumbashi said, “AI makes me look professional even when I’m working from my phone.”

#### 4.4.2 Sectoral applications

- *Agriculture*—AI-assisted crop-planning tools and weather-prediction apps.
- *Commerce*—Chatbots for customer engagement and product descriptions.
- *Education*—AI-assisted tutoring and translation.
- *Creative industries*—Graphic design, video scripting, and content generation.

#### 4.4.3 Early formalization pathways

AI helps youth:

- Standardize communication
- Improve customer trust
- Maintain digital records
- Present more professional branding

These practices contribute to *incremental formalization*, consistent with informal economy literature (Meagher, 2018).

### 4.5 Drivers and barriers within the socio-technical framework

To synthesize the empirical findings, a drivers-and-barriers framework is employed as a secondary interpretive lens to consolidate patterns emerging from the four socio-technical pillars. Drivers capture youth-led innovation capacities, including adaptive AI use, peer-based learning, and rapid experimentation, while barriers reflect persistent infrastructural constraints, high connectivity costs, limited formal training, and fragmented governance arrangements.

Used in this way, the drivers-and-barriers synthesis complements the thematic analysis by providing a concise overview of the enabling and constraining conditions shaping AI adoption in the informal economy.

Table 1 provides a high-level synthesis of the key drivers and barriers shaping youth-led AI adoption identified across the empirical material. It is intended as an analytical abstraction rather than an exhaustive inventory of individual practices.

**Table 1:** Drivers and barriers shaping youth-led ai adoption in the informal economy.

Dimension	Key empirical insights
Drivers	Youth adaptability (e.g., rapid experimentation with ChatGPT and mobile chatbots); peer-learning networks (e.g., WhatsApp groups and YouTube tutorials); low-cost, mobile-based AI use
Barriers	High data costs are limiting sustained AI use; unreliable electricity is disrupting work and learning; limited formal AI training; fragmented governance and regulatory uncertainty

## 5 Discussion

This section interprets the findings through ICT4D, socio-technical systems theory, and the capability approach, situating the DRC within broader Global South dynamics. The study shows that generative AI functions not only as a productivity tool but as an accelerator of capability formation under structural constraint. As illustrated in Figure 1, AI adoption follows a hybrid trajectory in which bottom-up, youth-led innovation intersects unevenly with top-down institutional frameworks, challenging linear, infrastructure-first models of digital transformation (Avgerou, 2008; Heeks, 2022).

The findings demonstrate that youth mobilize AI to bypass traditional skill acquisition pathways, enabling *compressed professionalization*: the rapid formation and immediate enactment of professional-level capabilities outside formal systems. Participants report using AI for tasks such as coding, digital marketing, and content creation, reflecting accelerated enactment of professional practices in informal contexts.

At a theoretical level, this reframes AI adoption as a process of temporal restructuring of learning and work. Generative AI reduces the lag between knowledge acquisition and application through mechanisms such as real-time cognitive scaffolding, iterative feedback loops, and access to generalized problem-solving templates. AI thus transforms not only what users can do, but how quickly and under what conditions they can do it.

However, this process is uneven. The findings support AI as a *conditional capability amplifier*, expanding agency while reproducing structural inequalities (Sen, 1999). Unequal access to connectivity, affordability, and sustained usage produces differentiated capability trajectories. AI therefore reconfigures, rather than removes, constraints by shifting inequality toward the depth and continuity of engagement.

These dynamics extend ICT4D by demonstrating non-linear pathways of capability formation, where youth-led improvisation precedes institutional alignment (Walsham, 2017; Heeks, 2022). At the same time, the findings engage AI scholarship by showing that generative AI reshapes expertise, enabling users to perform tasks associated with formal professionals, often at a surface level (Heeks, 2022). This raises concerns about deskilling, over-reliance, and limited depth of learning.

Finally, reliance on external AI platforms introduces issues of platform dependency and data colonialism, embedding local capability expansion within asymmetrical global systems (Toyama, 2015; Srnicek, 2017; Couldry and Mejias, 2019; Gwagwa et al., 2022).

Overall, AI adoption in informal economies emerges as a hybrid socio-technical process, shaped by the interaction of youth agency, infrastructural constraints, and weak institutional mediation, rather than as a purely transformative or deterministic force (Sumberg et al., 2024).

### 5.1 Infrastructure as a structural constraint

Infrastructure remains the most significant constraint on meaningful AI adoption. High data costs, unstable bandwidth, and unreliable electricity limit the frequency, complexity, and scalability of AI use. These constraints align with broader regional patterns linking AI readiness to affordable connectivity and resilient infrastructure (International Telecommunication Union, 2024; Organisation for Economic Co-operation and Development, 2023).

In the DRC, infrastructural scarcity not only restricts access but also shapes usage patterns, favoring lightweight, mobile-based applications over more advanced tools. As illustrated in Figure 1, this reflects persistent tensions in which policy ambitions outpace the infrastructural conditions required to support grassroots AI practices, reinforcing non-linear pathways of capability formation.

### 5.2 Governance fragmentation and institutional weakness

Governance gaps—including fragmented regulatory frameworks, limited coordination, and inconsistent policy implementation—continue to constrain AI adoption. Although national strategies articulate ambitious objectives, their operationalization remains limited by weak institutional capacity and the absence of structured mechanisms for youth participation.

Consistent with socio-technical systems theory, these findings highlight that technological outcomes depend on alignment between institutional arrangements and user practices (Avgerou, 2008; Orlikowski and Iacono, 2001). However, they also demonstrate that innovation can advance despite weak institutional mediation, producing partial and uneven alignment between policy and practice.

### 5.3 Skills ecosystems and informal learning pathways

Youth rely extensively on informal learning ecosystems—such as YouTube, WhatsApp, and peer networks—to acquire AI-related skills. These decentralized pathways enable rapid diffusion of basic competencies but remain limited in supporting advanced expertise and formal certification.

This pattern reinforces the capability approach, which emphasizes that technology expands agency only when supported by meaningful learning opportunities (Sen, 1999; Alkire, 2002). At the same time, it extends this perspective by showing that informal learning can enable immediate capability enactment, even in the absence of formal systems. As illustrated in Figure 1, this reflects persistent misalignment between policy ambitions and educational capacity.

### 5.4 AI as a catalyst for early formalization and diversification

Despite structural constraints, AI enables the emergence of hybrid microenterprises combining informal practices with digital tools. Use cases include AI-assisted marketing, chatbot-based customer engagement, automated inventory management, and translation services. These activities support incremental pathways toward formalization by enhancing visibility, trust, and operational structure (Meagher, 2018).

However, without improvements in infrastructure, governance, and skills ecosystems, such innovations risk remaining localized rather than scaling into broader transformation. Conceptualizing AI as a conditional capability amplifier highlights how expanded agency coexists with stratified inclusion shaped by access and usage conditions.

### 5.5 Policy–practice misalignment and institutional mediation

The findings reveal a persistent misalignment between national AI strategies and everyday youth practices. While policy frameworks emphasize formal training and institutional innovation ecosystems, AI adoption is primarily driven by informal, peer-based, and mobile-first experimentation (Heeks, 2022; Walsham, 2017).

Compared to more institutionalized ecosystems (e.g., Kenya, Rwanda), AI adoption in the DRC remains weakly mediated by the state. This highlights how institutional capacity shapes both the scale and modes of AI diffusion (Avgerou, 2008; Foster and Heeks, 2013). Persistent infrastructural constraints further reinforce bottom-up pathways while limiting scalability (International Telecommunication Union, 2024; Organisation for Economic Co-operation and Development, 2023).

These dynamics align with critiques of techno-solutionism, emphasizing amplification rather than structural transformation (Toyama, 2015). Effective AI governance in informal economies therefore requires adaptive institutional approaches that recognize and integrate youth-led innovation within broader policy frameworks (Gwagwa et al., 2022; Plantinga et al., 2024).

## 6 Strategic action framework

Building on the empirical findings and the four socio-technical pillars, this section proposes an integrated Strategic Action Framework to support equitable and scalable AI adoption in informal economies.

The framework consists of four pillars:

1. Digital Infrastructure and Equitable Access
2. Skills, Training, and Employability
3. Governance, Ethics, and Regulation
4. Innovation Ecosystems and Regional Cooperation

Each pillar identifies the socio-technical conditions required to scale youth-led AI adoption.

### 6.1 Pillar 1: digital infrastructure and equitable access

To enable meaningful AI adoption, the DRC must prioritize:

- Expansion of affordable broadband and mobile data services
- Investment in reliable electricity infrastructure
- Development of shared digital facilities (community labs, innovation hubs)
- Incentives for private-sector investment in connectivity

These measures reduce friction for youth innovators and expand the range of feasible AI applications.

### 6.2 Pillar 2: skills, training, and employability

A robust skills ecosystem requires:

- Structured digital-skills programs targeting youth
- Integration of AI literacy into secondary and tertiary education
- Partnerships between universities, tech hubs, and private firms
- Certification pathways for informal learners

These interventions support youth capability expansion and enable progression from basic AI use to more advanced competencies.

### 6.3 Pillar 3: governance, ethics, and regulation

Effective AI governance in the DRC should focus on:

- Strengthening inter-ministerial coordination
- Developing clear regulatory frameworks for AI use
- Ensuring ethical, inclusive, and context-appropriate standards
- Creating mechanisms for youth participation in policy design

These measures create enabling institutional environments for youth-led adoption and reduce uncertainty.

### 6.4 Pillar 4: innovation ecosystems and regional cooperation

To scale youth-led innovation, the DRC should:

- Support local tech hubs and community innovation centers
- Expand access to financing for youth-led digital enterprises
- Promote regional knowledge exchange through the African Union and SADC platforms
- Leverage diaspora expertise and investment

These interventions connect youth innovators to markets, capital, and networks, strengthening the broader innovation ecosystem. The Strategic Action Framework is intended as a heuristic guide grounded in the study’s empirical insights rather than a detailed policy blueprint. While developed in the DRC context, these recommendations apply to other informal economies characterized by structural informality and weak institutional mediation.

### 6.5 Limitations

This study has several limitations. First, its focus on urban, digitally engaged youth introduces potential sampling bias, with rural and less connected populations underrepresented. Second, the sample may over represent early adopters of AI, potentially skewing findings toward more optimistic adoption patterns. Third, the cross-sectional design limits the assessment of the long-term sustainability of AI-enabled micro-enterprises. Finally, the qualitative approach prioritizes depth and contextual understanding over statistical generalizability, consistent with the study’s conceptually driven orientation.

## 7 Conclusion

This article has examined how artificial intelligence (AI) is adopted and appropriated within informal economies in the Democratic Republic of the Congo (DRC), demonstrating that AI adoption is fundamentally a socio-technical process shaped by the interaction of youth agency, infrastructural constraints, and weak institutional mediation. Rather than unfolding through linear, policy-driven pathways, AI adoption emerges through bottom-up, improvisational practices embedded in everyday economic activity (Avgerou, 2008; Walsham, 2017).

The study makes three contributions. First, it introduces the concept of *compressed professionalization* to explain how generative AI enables the accelerated formation and immediate enactment of professional-level capabilities outside formal institutional pathways. This concept captures a shift from gradual skill acquisition to real-time capability deployment under conditions of structural constraint (Heeks, 2022; Sen, 1999).

Second, the analysis develops a process-based account of AI as a *conditional capability amplifier*, showing how mechanisms such as cognitive scaffolding, iterative feedback, and reduced learning-to-application lag reshape the relationship between learning, work, and economic participation. While AI expands agency, these effects remain uneven and contingent on access to infrastructure, skills, and sustained usage (Sen, 1999; Heeks, 2022).

Third, the study advances a non-linear, socio-technical model of AI adoption in informal economies, demonstrating that bottom-up innovation can precede and outpace institutional alignment. This challenges dominant infrastructure and policy-first models of digital transformation and highlights the central role of youth-led innovation in shaping emerging digital economies (Avgerou, 2008; Heeks, 2022).

Beyond the DRC context, these findings contribute to broader debates in ICT4D and AI scholarship by showing that generative AI is not only a technological tool but a structural force that reshapes the temporal dynamics of capability formation and economic participation. At the same time, the findings underscore the persistence of structural inequalities, as AI-enabled opportunities remain unevenly distributed and embedded within global systems characterized by platform dependency and asymmetrical value extraction (Toyama, 2015; Srnicek, 2017; Couldry and Mejias, 2019; Gwagwa et al., 2022).

From a policy perspective, the results suggest that effective AI governance in informal economies requires moving beyond top-down approaches toward adaptive frameworks that recognize and integrate existing youth-driven practices. Supporting equitable AI adoption will therefore depend not only on expanding infrastructure and formal training systems, but also on engaging with informal learning ecosystems and locally embedded innovation dynamics (Plantinga et al., 2024; Gwagwa et al., 2022).

Future research should examine the longitudinal trajectories of AI-enabled microenterprises, explore comparative dynamics across different institutional contexts, and further investigate how global AI infrastructures are locally appropriated and transformed. Understanding these dynamics is critical to ensuring that AI does not merely reproduce existing inequalities but instead contributes to more inclusive and context-sensitive pathways of digital transformation.

## Data Availability

The raw data supporting the conclusions of this article will be made available by the authors, without undue reservation.
